# Performance of methods for analyzing continuous data from stratified cluster randomized trials – A simulation study

**DOI:** 10.1016/j.conctc.2023.101115

**Published:** 2023-03-14

**Authors:** Sayem Borhan, Jinhui Ma, Alexandra Papaioannou, Jonathan Adachi, Lehana Thabane

**Affiliations:** a Department of Health Research Methods, Evidence, and Impact, McMaster University, Hamilton, ON, Canada; b Biostatistics Unit, Research Institute of St Joseph's Healthcare, Hamilton, ON, Canada; c GERAS Centre, Hamilton Health Sciences, Hamilton, ON, Canada; d Department of Medicine, McMaster University, Hamilton, ON, Canada

**Keywords:** Cluster randomized trials, Stratified design, Simulation, Continuous

## Abstract

**Background:**

The adoption of cluster randomized trials (CRTs) with the stratified design is currently gaining widespread interest. In the stratified design, clusters are first grouped into two or more strata and then randomized into treatment groups within each stratum. In this study, we evaluated the performance of several commonly used methods for analyzing continuous data from stratified CRTs.

**Methods:**

This is a simulation study where we compared four methods: mixed-effects, generalized estimating equation (GEE), cluster-level (CL) linear regression and meta-regression methods to analyze the continuous data from stratified CRTs using a simulation study with varying numbers of clusters, cluster sizes, intra-cluster correlation coefficients (ICCs) and effect sizes. This study was based on a stratified CRT with one stratification variable with two strata. The performance of the methods was evaluated in terms of the type I error rate, empirical power, root mean square error (RMSE), and width and coverage of the 95% confidence interval (CI).

**Results:**

GEE and meta-regression methods had high type I error rates, higher than 10%, for the small number of clusters. All methods had similar accuracy, measured through RMSE, except meta-regression. Similarly, all methods but meta-regression had similar widths of 95% CIs for the small number of clusters. For the same sample size, the empirical power for all methods decreased as the value of the ICC increased.

**Conclusion:**

In this study, we evaluated the performance of several methods for analyzing continuous data from stratified CRTs. Meta-regression was the least efficient method compared to other methods.

## 1 Background

There is an increasing trend in adopting cluster randomized trials (CRTs) for assessing the treatment effect [1], where intact clusters of individuals, rather than individual participants, are randomized into treatment groups [2]. The types of clusters can be distinct, including geographical regions [3], healthcare areas [4], and schools [5]. Three designs are commonly used to allocate clusters into treatment groups: (i) completely randomized: involves no stratification or matching of clusters; (ii) matched-pair: involves matching of two clusters and then random assignment to treatment groups within each pair; and (iii) stratified: involves the random allocation of more than two clusters into treatment groups within each stratum [2]. For example, Mallick et al. [5] conducted a school-based stratified CRT, where schools were first divided into quintiles (1–3: lower and 4–5: higher) based on socio-economic resources and then stratified into low versus high quintiles. Schools within each stratum were then randomly allocated to treatment and control groups [5].

The implementation of a stratified design to allocate clusters, which leads to a more efficient design [6], is increasing in CRTs [7]. Like randomized controlled trials (RCTs) on individuals, this design helps to achieve balance among the treatment arms within each stratum. The stratified design falls between completely randomized and matched-pair design and involves a fewer number of strata than the matched-pair design [8].

In CRT, there might be similarities among the outcomes from the same cluster [2]. This similarity or clustering is measured through the intra-cluster correlation coefficient (ICC), and statistical methods should adjust for this clustering [2]. Failure to account for this clustering may lead to false significance of the treatment effect [2,9]. Furthermore, we need to calculate the sample size for CRT by considering the desired power, effect sizes, and ICC. Thus, the power to detect the treatment effect depends on the accounting of ICC, effect size and sample size [10]. Moreover, a majority of the literature suggested that the stratification variable(s) used in the randomization should be adjusted for in the analysis [2,8,[11], [12], [13], [14]], while a few studies argued in favour of non-adjustment [15]. Studies based on both stratified RCTs on individuals and stratified CRTs indicated that the failure to adjust for stratification leads to wider confidence intervals, larger p-values and a reduction in power [[16], [17], [18]]. Thus, ignoring the adjustment for both clustering and stratification may yield a misleading conclusion about the treatment effect, and society may miss out on the benefit of a treatment.

A recent review conducted by our team indicated that only 38% of the studies adjusted the primary method for both clustering and stratification for assessing the treatment effect [7]. Individual-level (based on individual-level data) and cluster-level (based on the cluster-level summary) methods can be used to examine the effect of treatment from the stratified CRTs. The individual-level methods include mixed-effects [19] or generalized estimating equation (GEE) [20] methods, while cluster-level methods include cluster-level linear regression or meta-analytic approach [21,22] – which can be used to assess the treatment effect over strata. Thompson et al. [23] demonstrated the use of the meta-analysis technique for analyzing matched-pair CRTs.

Researchers have investigated the performance of methods for analyzing data from CRTs [[24], [25], [26], [27]]. Klar and Darlington [24] studied the performance of several mixed-effects methods to analyze the pretest-posttest continuous data from the completely randomized CRTs. They suggested that it is possible to gain power by adding the individual and cluster-level associations between the baseline and follow-up measurements in the model. Moerbeek et al. [25] compared the performance of naïve regression, fixed-effects regression, linear regression based on summary measure and multilevel regression methods in terms of the estimated standard error of the treatment effect and suggested to use multilevel regression in the case of CRT or multi-centre trials. The authors also demonstrated the equivalency between the regression model based on summary measure and the mixed-effects method [25]. On the other hand, Austin [26] investigated the performance of methods for analyzing binary data from the CRTs. He found that in most scenarios, there was a negligible difference among the methods, while the GEE method yielded higher power. Leyrat et al. [27] conducted a simulation study and compared the performance of twelve analysis methods for analyzing continuous data from the CRTs with 40 or fewer number of clusters. The authors recommended small sample corrections - degrees of freedom correction for mixed-effects method and standard error (SE) correction for GEE, for analyzing continuous data from CRTs with the small number of clusters, while van Breukelen and Candel [28] provided a mathematical explanation of their findings. Borhan et al. [17,18] empirically compared several methods for analyzing continuous and count data from stratified CRTs using the data from the Mallick et al. [5] and the ViD*OS* study [29], respectively. In these studies, the authors found that the overall conclusions in terms of statistical significance were similar for all methods. However, these methods differed in terms of the estimated treatment effect and the precision. Furthermore, since these studies were based on empirical data, it is challenging to draw conclusions about the statistical properties of these methods. There is literature available that demonstrated the performance of methods for analyzing different types of data from the CRTs. However, most of the studies were based on completely randomized CRTs, and we still lacked evidence regarding the performance of methods for analyzing continuous data from the stratified CRTs.

In this study, we conducted a simulation study to examine the performance of methods for assessing the treatment effect from stratified CRTs. We evaluated several methods in terms of type I error rate, empirical power, root mean square error rate, and width and coverage of the 95% confidence intervals, for analyzing continuous data from the stratified CRTs.

## 2 Methods

This was a simulation study where we evaluated the performance of several methods for assessing the treatment effect from stratified CRTs when the outcome of interest was continuous.

## 3 Patient and public involvement

Patient and public involvement are not applicable for this study.

## 4 Statistical methods

Both individual-level and cluster-level methods were used to assess the treatment effect. These methods were adjusted for stratification. All of these methods assumed that there was no interaction between the treatment and the stratification variable.

## 5 Individual-level methods

### 5.1 Mixed-effects regression model (mixed-effects)

The mixed-effects regression model is given by

(1) $$Y_{ijks}=\beta_{01}+\beta_{11}X_{ijks}+\beta_{21}S_{ijks}+C_{.jk}+e_{ijks}$$

where $Y_{ijks}$ is the outcome of the i-th subject in the j-th cluster, k-th treatment group and s-th stratum. $X_{ijks}$ represents the treatment assignment ($X_{ijks}$ = 1 for the treatment group; $X_{ijks}$ = 0 for the control group), $S_{ijks}$ represents the dichotomous stratification variable with value 0 and 1, and $e_{ijks}$ is the random error term assumed to follow a normal distribution with mean 0 and variance $\sigma_{e}^{2}$.

In this model, $\beta_{01}$ represents the expected outcome in the control group for the stratum 0, while $\beta_{11}$ and $\beta_{21}$ represents the treatment and stratum effect, respectively, which are fixed. The random cluster effect is given by $C_{.jk}$, which follows a normal distribution with mean 0 and variance $\sigma_{b}^{2}$. The ICC represents the correlation between two randomly chosen subjects in the same cluster. A single common ICC is given by $\frac{\sigma_{b}^{2}}{\sigma_{b}^{2}+\sigma_{e}^{2}}$ and was assumed equal for all clusters. The lmer () function of the R package lmerTest() was used to fit this model with the restricted maximum likelihood (REML) method [30]. The treatment effect was assessed using the t-test, and the Satterthwaite method – based on the first two moments of the estimated parameter, was used to calculate the degrees of freedom [31].

### 5.2 Generalized estimating equation (GEE)

The generalized estimating equation (GEE) model is given by

(2) $$g\left(\mu_{ijks}\right)=\beta_{02}+\beta_{12}X_{ijks}+\beta_{22}S_{ijks}$$

where, ${\mu_{ijks}=E(Y}_{ijks})$

Like the mixed-effects model, $\beta_{02}$ represents the population-averaged value of the outcome in the control group for the stratum 0, while $\beta_{12}$ and $\beta_{22}$ represent the treatment and the stratum effect, respectively. The working correlation structure in the GEE model takes into account the correlation among the outcomes from the same cluster, and the sandwich covariance estimator yields a robust estimate of the treatment effect, even if the correlation structure is misspecified [32]. It is notable that, the model-based estimation of the standard error of the treatment effect is not robust to the misspecification of the correlation structure. In the GEE analysis, we assumed the correlation structure followed an exchangeable pattern. The exchangeable pattern assumes the correlations among the outcomes from the individuals within the same cluster are constant. R package geepack() was used to fit the GEE model. The Chi-square test was used to assess the treatment effect, which follows a chi-square distribution with 1° of freedom.

## 6 Cluster-level methods

### 6.1 Cluster-level linear regression (CL linear regression)

The cluster-level method is based on cluster-level summary measures, such as the mean [2]. We first calculated the mean for each cluster, and then a linear regression was fitted and adjusted for stratification, using these means. Cluster-level linear regression is given by:

(3) $${\bar{Y}}_{.jks}=\beta_{03}+\beta_{13}X_{.jks}+\beta_{23}S_{.jks}+e_{.jks}$$

where $\beta_{03}$ represents the expected outcome in the control group for the stratum 0, while $\beta_{13}$ and $\beta_{23}$ represents the treatment and stratum effect, respectively. The treatment effect was assessed using the t-test, which follows a t distribution with n−p−1 degrees of freedom, where n is the number of clusters and p(=3) is the number of estimated parameters.

### 6.2 Meta-regression

The meta-regression approach is based on summary measures [21]. First, we estimated the mean for each cluster by treatment group in each stratum. Then we calculated the mean of these cluster means by treatment group in each stratum. After that, we estimated the mean difference between the treatment and control groups in each stratum. Then we used the fixed-effects model to estimate the treatment effect, which was performed using the R package metafor(). Let ${\hat{\beta }}_{s}$ is the estimated treatment effect for the s-th stratum. The random-effects meta-regression model is given by [21]

(4) $${\hat{\beta }}_{s}=\beta +\varepsilon_{s}$$

where β is the overall mean effect size; $\varepsilon_{s}$ is the error term. The error terms $\varepsilon_{s}$ follows a normal distribution with mean 0 and variance $\sigma_{sb}^{2}$ i.e. $\varepsilon_{s}∼N\left(0,\sigma_{sb}^{2}\right)$, where $\sigma_{sb}^{2}$ is the within stratum variance. We chose the fixed-effects model because there is only one stratification variable with two strata. The treatment effect was assessed using the z-test, which follows a standard normal distribution.

### 6.3 Simulation study design

The simulation study was designed using the approach adopted by Arnold et al. [33] and Moerbeek and Schie [34]. We considered a stratified design with one stratification variable with two strata. The outcome, Y, was generated using the following mixed-effects linear regression model: $Y_{ijks}=\beta_{0}+\beta_{1}X_{ijks}+\beta_{2}S_{ijks}+C_{.jk}+e_{ijks}$; where, $Y_{ijks}$ is the outcome of the i-th subject in the j-th cluster, in the k-th treatment group and s-th stratum; $X_{ijks}\left(=0,1\right)$ represent the dummy variable for treatment allocation ($i=1,\ldots ,n_{j};j=1,\ldots ,J,s=0,1$); $C_{.jk}$ is the cluster-level random effect while $e_{ijks}$ is the individual-level random error term. Both $C_{.jk}$ and $e_{ijks}$ follow normal distributions with mean 0 and standard deviations $\sigma_{b}$ and $\sigma_{e}$, respectively. Random effects and error term related to the ICC as ICC ($=\frac{\sigma_{b}^{2}}{\sigma_{b}^{2}+\sigma_{e}^{2}}$) is the ratio between cluster variance to the total variance [2]. Without loss of generality, the total variability was fixed at $\sigma^{2}=\sigma_{b}^{2}+\sigma_{e}^{2}=1$ and $\beta_{0}=0$. The other parameters for this simulation study such as, the number of clusters, cluster sizes, treatment effect sizes, and ICCs, were selected, given in Table 1, based on the studies that had a continuous outcome as the primary outcome from our recently conducted systematic survey [7]. The estimated number of simulations was 2500, which can produce an estimate within 5% accuracy of the true treatment effect of 0.11 with a 5% level of significance, assuming the variance of the estimate was 0.0096 and with 80% power. So, 2500 simulations were run for each combination of $\beta_{1}=0,0.11,0.50$; $\beta_{2}=0.11,0.50$, number of clusters per stratum = 6, 24, 34, 68; number of individuals per cluster = 5,10,15,20,25,30,35,40,45,50 and ICC = 0.03, 0.06, 0.10. The treatment effect $\beta_{1}=0.11$ and $\beta_{1}=0.50$ indicated the low and medium effect, respectively. The number of clusters in each stratum was equally divided into treatment groups within each stratum. This simulation study was conducted using R [35].

**Table 1:** Parameters for the simulation study.

Variable	Summary from studies that had continuous outcome as their primary outcome^a^	Selected for simulation study
Number of clusters in the treatment groups	Mean = 50; Median = 34; Q1 = 24; Q3 = 68; Min = 6; Max = 228	Total number of clusters per stratum: 6, 24, 34, 68
Number of individuals per cluster	–	5, 10, 15, 20, 25, 30, 35, 40, 45, 50
Number of stratification variables	Mean = 2; Median = 1; Q1 = 1; Q3 = 2; Min = 1; Max = 3	1
Effect size	Mean = 0.36; Median = 0.11; Q1 = −0.12; Q3 = 1.02; Min = −9.12; Max = 7.17	0, 0.11, 0.50
ICC	Mean = 0.09; Median = 0.03; Q1 = 0.00; Q3 = 0.06; Min = 0.00; Max = 0.58	0.03, 0.06, 0.10

Q1 = 25th percentile; Q3 = 75th percentile; ICC = intra-cluster correlation coefficient.

a Summary of these variables were calculated form our recently conducted systematic survey [7].

### 6.4 Comparison of methods

We applied the methods discussed in the statistical methods section to assess the treatment effect for each of the simulated data sets. The following quantities were used to evaluate the performance of these methods: (1) type I error rate was measured as the proportion of the time the test statistic rejected the null hypothesis of the treatment effect $H_{0}:\beta_{1}=0$, when the true treatment effect was $\beta_{1}=0$, out of 2500 replications; (2) empirical power was measured as the proportion of the time the test statistic rejected the null hypothesis of the treatment effect $H_{0}:\beta_{1}=0$, when the true treatment effect was $\beta_{1}=0.11$ or 0.50, out of 2500 replications; (3) root mean square error (RMSE) was measured as the $\sqrt{E\left[{\left(\hat{\beta_{1}}-\beta_{1}\right)}^{2}\right]}$, where $\hat{\beta_{1}}$ and $\beta_{1}$ are the estimated and the true value of the treatment effect; (4) average width of the 95% CIs was measured as the mean of the difference between the upper limit and the lower limit across all 2500 replications; (5) empirical coverage was measured as the percentage of the time the 95% confidence intervals (CIs) contain the true value of the treatment effect over 2500 replications;

### 6.5 Comparison of methods using example data

We also compared the above-mentioned methods to assess the treatment effect from Mallick et al. study [36]. This study conducted a stratified cluster randomized trial to examine the impact of Classroom Communication Resource (CCR) on peer attitude toward Children Who Stutter (CWS) in South African schools in the Western Cape; details can be found elsewhere [5,15]. The schools were stratified into high or low-quintile groups and then randomized into CCR or usual care groups within each stratum. The primary outcome was Stuttering Resource Outcomes Measure (SROM), measured at baseline and 6-month post-treatment.

## 7 Results

### 7.1 Type I error rate

**Fig. 1:**
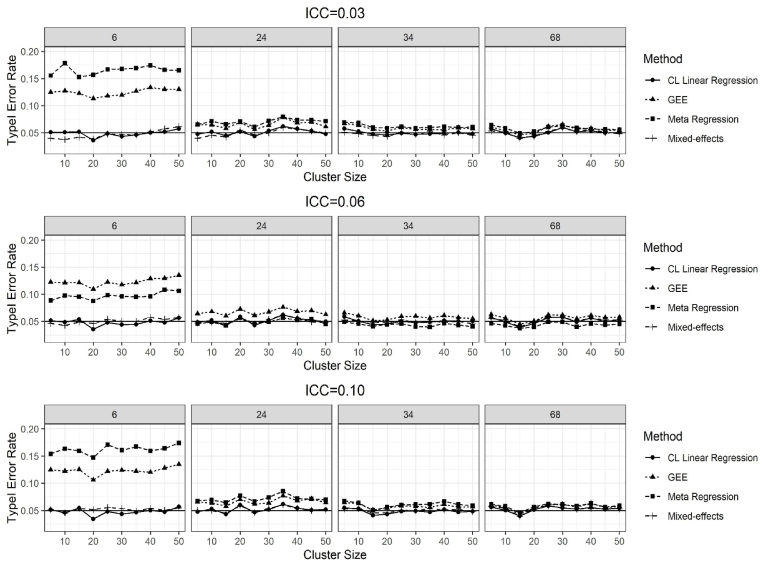
Results of the type I error rate for testing the H_0 of no treatment effect when the true treatment effect was 0, over 2500 simulations for the ICC = 0.03, 0.06, and 0.10 and the number of clusters per stratum was 6, 24, 34 and 68.

The results of the type I error rate for all methods are given in Fig. 1 for the ICC = 0.03, 0.06 and 0.10. The type I error rates were more than 10% for the GEE and the meta-regression methods when the number of clusters was 6 per stratum for all ICC values. The type I error rates decreased to around 6% when the number of clusters increased to 24 and 34 per stratum for the GEE and meta-regression methods (Fig. 1). Moreover, both methods yielded type I error rates of around 5% for the number of clusters 68 per stratum. The CL linear regression and mixed-effects methods yielded type I error rates around the nominal 5% for all combinations of the number of clusters and the cluster sizes (Fig. 1). All methods followed a similar pattern for the ICC values of 0.03, 0.06 and 0.10 (Fig. 1). There was very little difference in type I error rates when the stratum effect was 0.50 (Table A1 in the supplemental materials).

We excluded the number of clusters 6 for further comparison as GEE and meta-regression methods failed to yield type I error rates close to 5%.

### 7.2 Empirical power

**Fig. 2:**
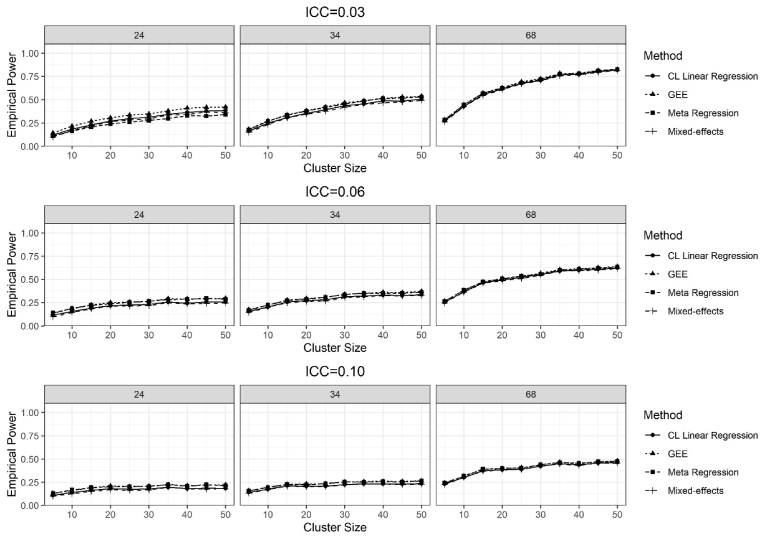
Results of the empirical power for testing the H_0 of no treatment effect, while the true treatment effect was 0.11, over 2500 simulations for the ICC = 0.03, 0.06, and 0.10 and the number of clusters per stratum was 24, 34, and 68.

**Fig. 3:**
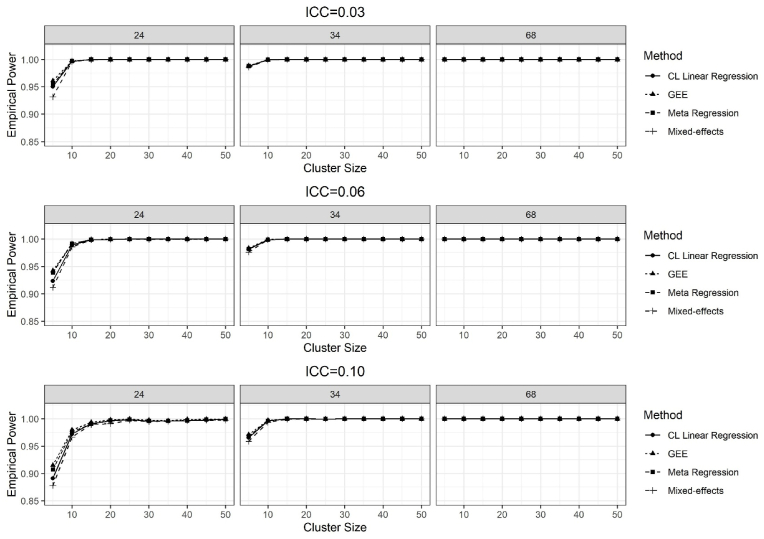
Results of the empirical power for testing the H_0 of no treatment effect, while the true treatment effect was 0.50, over 2500 simulations for the ICC = 0.03, 0.06, and 0.10 and the number of clusters per stratum was 24, 34, and 68.

The results of empirical power for all methods are provided in Fig. 2, Fig. 3 for the ICC = 0.03, 0.06 and 0.10, and for the treatment effects of 0.11 and 0.50, respectively, while the stratum effect was 0.11.

For the number of clusters 24 and ICC = 0.03, GEE had the highest power on average 32% across all number of individuals per cluster compared to other methods. For ICC = 0.06 and the number of clusters 24, meta-regression and GEE methods had slightly more power, around 25% on average across all number of individuals per cluster, while CL linear regression and mixed-effects methods had the power of around 21% (Fig. 2). All methods had almost similar power 65%, 51%, and 40%, on average, across all number of individuals per cluster, for ICC = 0.03, 0.06, 0.10 and the number of clusters 68 (Fig. 2) per stratum, respectively. Power for all methods increased as the number of individuals per cluster increased for all combinations of the number of clusters and ICCs. However, the power for all methods was decreased as the value of the ICC increased for all combinations of the number of clusters and cluster sizes. (Fig. 2).

The results of the empirical power for all methods for the treatment effect of 0.50 are given in Fig. 3. The power for all methods dramatically increased as the treatment effect increased to 0.50 from 0.11 for all combinations of the number of clusters, cluster sizes and the ICCs (Fig. 3). The power was similar when the treatment effect was 0.50, but the stratum effect was increased from 0.11 to 0.50 (Table A2 in the supplemental materials).

### 7.3 Root mean square error (RMSE)

**Fig. 4:**
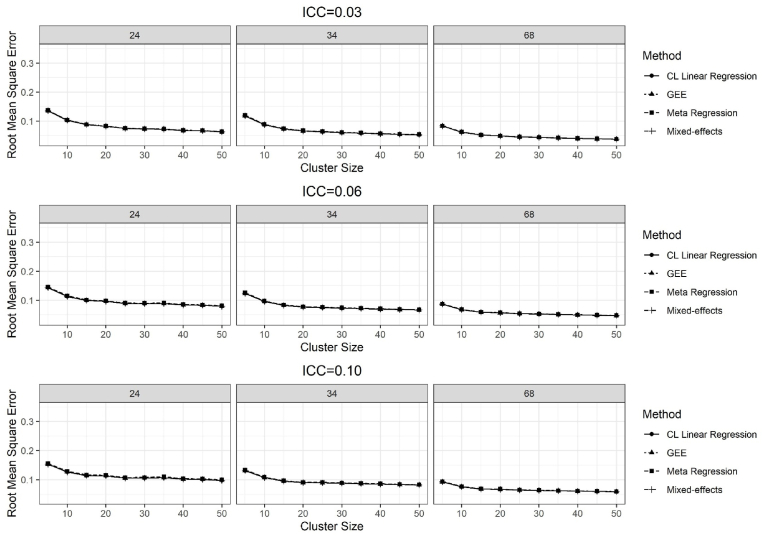
Results of the RMSE for the true treatment effect of 0.11, over 2500 simulations for the ICC = 0.03, 0.06, and 0.10, and the number of clusters per stratum was 24, 34, and 68.

The results of the average root mean square error for all methods for the treatment effect of 0.11 and for the ICC values of 0.03, 0.06 and 0.10 are given in Fig. 4.

Overall, the average RMSEs decreased as the number of clusters, and the cluster sizes increased. All methods had similar RMSEs, except the meta-regression, for all combinations of the cluster sizes and the number of clusters (Fig. 4). Meta-regression had slightly higher average RMSEs for the number of clusters 6 per stratum. The results of average RMSEs were almost similar when the treatment effect was 0.50, and the results were not presented here.

Width of the 95% confidence intervals.

**Fig. 5:**
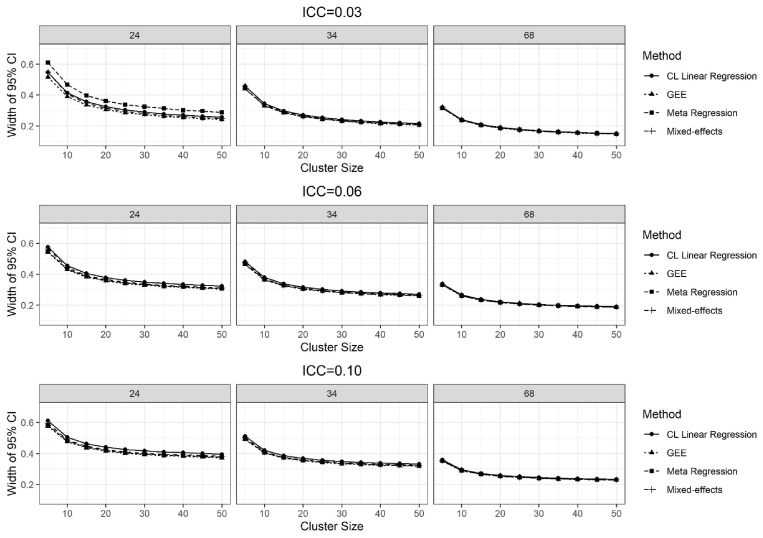
Results of the width of 95% CI for the true treatment effect of 0.11, over 2500 simulations for the ICC = 0.03, 0.06, and 0.10, and the number of clusters per stratum was 24, 34 and 68.

**Fig. 6:**
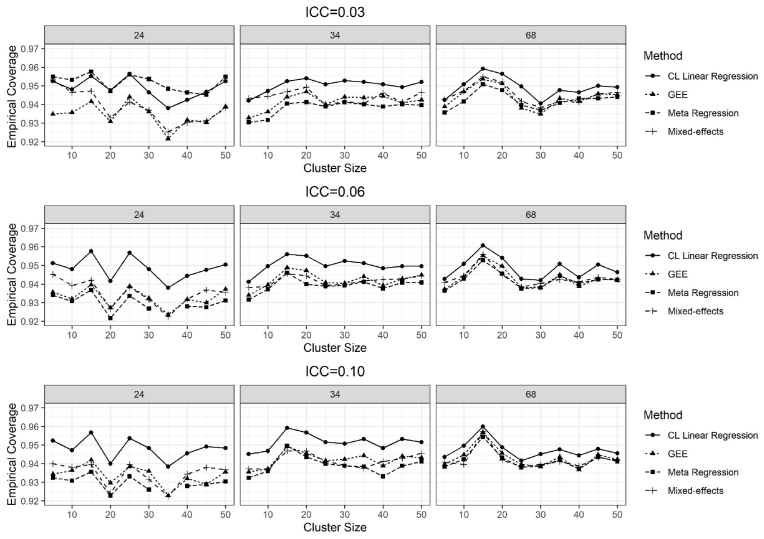
Results of the coverage for the true treatment effect of 0.11, over 2500 simulations for the ICC = 0.03, 0.06, and 0.10, and the number of clusters per stratum was 24, 34, and 68.

The results of the average widths of the 95% CIs for the treatment effect of 0.11 and the ICC values of 0.03, 0.06 and 0.10 are given in Fig. 6. For the number of clusters 24, meta-regression had the widest width, on average 38%, across all numbers of individuals. Overall, the cluster-level linear regression had slightly wider widths compared to other methods (except the number of clusters 24). Widths of the 95% CI increases as the ICC increases for all methods (Fig. 5).

### 7.4 Empirical coverage of the 95% confidence intervals

The results of the empirical coverage of the 95% CIs for the treatment effect of 0.11 and the ICC values of 0.03, 0.06 and 0.10 are given in Fig. 6. All methods had coverage of 94% or 95%, on average, for the number of clusters 34 and 68 (Fig. 6). GEE and meta-regression methods had slightly lower coverage, on average 93%, for the number of clusters 24 and when the ICCs were 0.06 and 0.10.

### 7.5 Results from example data

In total, the selected ten schools were stratified into two groups: higher quintile (6 schools) and lower quintile (4 schools). The schools were then randomized into the intervention CCR group and the control usual care group. The average cluster size was 45 (range: 30–54) and 46 (range: 18–68) in the CCR and usual care groups, respectively. Overall, 454 students (223 in the CCR group and 231 in the usual care group) participated in this study. The average age was 13 years for both groups.

The results of the estimated effect of the CCR obtained using the four methods are given in Table 2. We had a very small number of clusters (<10) per stratum. The overall conclusion from all the methods was similar; that is, there was no significant difference between CCR and usual care groups. The estimated effect sizes had the same direction for all methods except meta-regression. Cluster-level linear regression had the largest width of the 95% confidence interval, while meta-regression had the lowest width (Table 2). The widths of the 95% confidence interval for the GEE and the mixed-effects methods were almost similar. From the results of our simulation study, the average width of the 95% CI for the cluster-level linear was the highest for the number of clusters 6 per stratum and cluster size 45 (results are not presented here).

**Table 2:** Results for assessing the treatment (CCR^a^) effect using the data from the Mallick et al. study.

Method	Mean difference	95% Confidence Interval
GEE with Exchangeable	−0.10	(-1.47, 1.27)
Mixed-effects	−0.09	(-1.65, 1.46)
Cluster-level linear regression	−0.09	(-1.85, 1.68)
Meta-regression	0.08	(-0.99, 1.15)

a CCR: Classroom Curriculum Resource.

## 8 Discussion

In this study, we investigated the performance of several methods for assessing the treatment effect from the stratified CRTs with a single stratification variable. We have compared four different methods: GEE, mixed-effects, CL linear regression and meta-regression methods. It is evident that the number of clusters, cluster sizes, and ICCs had impacted the performance of these methods, evaluated through the type I error rate, empirical power, root mean square error and the width of 95% confidence intervals.

GEE and meta-regression methods yielded type I error rates of higher than 10% for the small number of clusters. On the other hand, CL linear regression and mixed-effects methods yielded satisfactory, approximately 5%, type I error rates. Borhan et al. [37] investigated the performance of methods for analyzing pretest-posttest binary data from completely randomized CRTs and found that the GEE method yielded liberal type I error rates for the small number of clusters. Similarly, Klar and Darlington [24] reported that mixed-effects methods yielded a satisfactory 5% type I error rate when the authors analyzed the continuous data from the completely randomized CRTs. These findings were in line with our findings.

The GEE method yielded higher empirical power compared to other methods for the small number of clusters, which matched with the findings of Austin [26], as the GEE method yielded more power compared to other methods when the author investigated the methods for analyzing binary data from CRTs. Researchers noticed that the sandwich covariance method underestimates the standard error of the treatment effect in the case of the small number of clusters, which inflated the type I error rate and empirical power [38,39]. Likewise, we found the average standard errors of the estimated treatment effect were lower for the GEE and the meta-regression methods than the mixed-effect and CL linear regression methods when the treatment effect was 0.11 (Table A3 in the supplemental materials).

The average RMSEs were similar for all methods except the meta-regression method. The GEE had similar average RMSEs as CL linear regression and mixed-effects methods due to inflated type I error rates for the small number of clusters. The meta-regression method yielded slightly higher RMSEs compared to other methods for the small number clusters, which was in line with the findings of Chu et al. [22]. The meta-regression method yielded the widest 95% confidence intervals, compared to other methods for a large number of clusters.

The simulation of this study yielded the predicted results and confirmed that the performance of all methods improved as the number of clusters, cluster sizes, and effect sizes increased. Also, for the same sample size, the performance of these methods worsened with the increased ICCs. Thus, this study reemphasized the need for correctly calculating the sample size to assess the treatment effect. More specifically, the sample size needed to be increased with the increased ICC, while we needed a smaller sample size with the increased effect size, as demonstrated by other researchers [10].

In this study, we compared methods utilizing individual-level (mixed-effects and GEE) and cluster-level summary (cluster-level linear regression and meta-regression) data. There is no possibility of losing any information for methods based on individual-level data, while this is not true for the methods based on cluster-level summary measures. From an inferential perspective, individual-level methods fall into two categories: (i) cluster-specific (e.g. mixed-effects method), which estimates the average intervention effect if a participant stays in the same cluster but moves from control to treatment arm; (ii) population-average (e.g. GEE method), which estimates the average intervention effect if a participant in the population moves from control to treatment arm [40]. Therefore, researchers need to consider this inferential difference between mixed-effects and GEE methods when examining the treatment effect.

There were several limitations of this study: first, we considered only one stratification variable with two strata. It is possible to extend this study by including more than two strata. In this case, the performance, especially type I error, and power, of the methods discussed in this study will be affected as assessing the treatment effect will require more degrees of freedom; second, we considered only 1:1 allocation of clusters into treatments groups and fixed cluster size in each stratum; third, this study was limited to a two-arm parallel-group trial; third, we compared the methods using the data from a real-life stratified CRT with very small number of clusters per stratum. finally, models used for data generation and analysis were based on the assumption that there was no interaction between treatment and stratification variable. From our systematic survey [7] we found assessing the interaction between treatment and stratification variable is very rare, partly because it requires more sample size. Incorporation of interaction will lower the degrees of freedom for testing the treatment effect and affect the type I error rate and power of the methods considered in this study.

This study shed light on the performance of methods – adjusted for stratification and clustering for analyzing continuous data from the stratified CRTs and would help researchers make an informed decision.

## 9 Conclusions

In this simulation study, we investigated the performance of four methods, adjusted for stratification and clustering, for analyzing the continuous data from the stratified cluster randomized trials with one stratification variable. The power for all methods improved as the number of clusters, and cluster sizes increased, while the power for all methods worsened as the ICC increased for the same sample size. GEE and meta-regression methods yielded type I error rates of higher than 10% for the small number of clusters. Meta-regression was the least efficient compared to other methods.

## Ethics approval and consent to participate

Not applicable.

## Consent for publication

Not applicable.

## Availability of data and materials

This was simulation study and no real data were used.

## Funding

There is no funding for this study.

## Authors’ contributions

SB and LT conceived the research question. SB conceptualized, developed and designed the study. LT contributed to the conceptualization and design of the study. SB performed the analyses, prepare the results and draft the manuscript. SB, JM, AP, JA and LT contributed equally to further improve this manuscript. All authors have read and approved the manuscript.

## Declaration of competing interest

The authors declare that they have no known competing financial interests or personal relationships that could have appeared to influence the work reported in this paper.

## List of abbreviation

CRT: Cluster Randomized Trial
GEE: Generalized Estimating Equation
CL: Cluster-level
CI: Confidence Interval
ICC: Intra-cluster Correlation Coefficient
REML: Restricted Maximum Likelihood
RMSE: Root Mean Square Error
