# Supplementary material for: Performance of methods for analyzing continuous data from stratified cluster randomized trials – A simulation study
Source: Contemp Clin Trials Commun. 2023 Mar 14;33:101115. doi: 10.1016/j.conctc.2023.101115 (PMC10313865; doi:10.1016/j.conctc.2023.101115)
Supplement: Multimedia component 1 [file mmc1.docx]

**Appendix: Supplemental materials**

**Table A1: Results of the type I error rate for the treatment effect=0 and stratum effect=0.50**

| **Number of clusters** | **Number of individuals per cluster** | **ICC=0.03** | | | | **ICC=0.06** | | | | **ICC=0.10** | | | |
| --- | --- | --- | --- | --- | --- | --- | --- | --- | --- | --- | --- | --- | --- |
|  |  | **CL Linear Regression** | **GEE** | **Meta-Regression** | **Mixed-effects** | **CL Linear Regression** | **GEE** | **Meta-Regression** | **Mixed-effects** | **CL Linear Regression** | **GEE** | **Meta-Regression** | **Mixed-effects** |
| **6** | 5 | 0.051 | 0.125 | 0.156 | 0.040 | 0.052 | 0.122 | 0.155 | 0.046 | 0.051 | 0.125 | 0.154 | 0.052 |
|  | 10 | 0.051 | 0.127 | 0.178 | 0.038 | 0.049 | 0.121 | 0.175 | 0.043 | 0.046 | 0.122 | 0.164 | 0.046 |
|  | 15 | 0.052 | 0.123 | 0.153 | 0.042 | 0.054 | 0.122 | 0.156 | 0.049 | 0.054 | 0.126 | 0.160 | 0.054 |
|  | 20 | 0.036 | 0.113 | 0.157 | 0.039 | 0.036 | 0.110 | 0.152 | 0.046 | 0.034 | 0.106 | 0.147 | 0.052 |
|  | 25 | 0.048 | 0.118 | 0.167 | 0.048 | 0.048 | 0.122 | 0.170 | 0.054 | 0.048 | 0.122 | 0.171 | 0.055 |
|  | 30 | 0.043 | 0.120 | 0.168 | 0.047 | 0.044 | 0.118 | 0.162 | 0.050 | 0.044 | 0.124 | 0.160 | 0.054 |
|  | 35 | 0.046 | 0.127 | 0.169 | 0.046 | 0.044 | 0.122 | 0.169 | 0.050 | 0.046 | 0.122 | 0.167 | 0.049 |
|  | 40 | 0.050 | 0.134 | 0.174 | 0.052 | 0.051 | 0.129 | 0.166 | 0.058 | 0.050 | 0.120 | 0.160 | 0.054 |
|  | 45 | 0.052 | 0.130 | 0.166 | 0.057 | 0.048 | 0.130 | 0.167 | 0.054 | 0.047 | 0.128 | 0.164 | 0.050 |
|  | 50 | 0.058 | 0.130 | 0.165 | 0.062 | 0.056 | 0.135 | 0.171 | 0.057 | 0.057 | 0.135 | 0.174 | 0.055 |
| **24** | 5 | 0.048 | 0.065 | 0.065 | 0.040 | 0.049 | 0.064 | 0.066 | 0.047 | 0.048 | 0.066 | 0.068 | 0.050 |
|  | 10 | 0.052 | 0.064 | 0.071 | 0.046 | 0.052 | 0.068 | 0.069 | 0.049 | 0.053 | 0.064 | 0.069 | 0.050 |
|  | 15 | 0.045 | 0.058 | 0.066 | 0.042 | 0.042 | 0.060 | 0.063 | 0.046 | 0.043 | 0.058 | 0.064 | 0.045 |
|  | 20 | 0.052 | 0.069 | 0.071 | 0.054 | 0.058 | 0.073 | 0.078 | 0.055 | 0.060 | 0.070 | 0.077 | 0.059 |
|  | 25 | 0.044 | 0.056 | 0.061 | 0.046 | 0.043 | 0.061 | 0.066 | 0.045 | 0.046 | 0.062 | 0.067 | 0.046 |
|  | 30 | 0.054 | 0.064 | 0.072 | 0.050 | 0.052 | 0.068 | 0.073 | 0.054 | 0.052 | 0.064 | 0.074 | 0.054 |
|  | 35 | 0.062 | 0.078 | 0.080 | 0.060 | 0.062 | 0.076 | 0.082 | 0.062 | 0.062 | 0.077 | 0.085 | 0.060 |
|  | 40 | 0.058 | 0.068 | 0.074 | 0.057 | 0.056 | 0.068 | 0.072 | 0.056 | 0.054 | 0.068 | 0.072 | 0.054 |
|  | 45 | 0.053 | 0.070 | 0.074 | 0.053 | 0.052 | 0.070 | 0.072 | 0.049 | 0.051 | 0.071 | 0.071 | 0.050 |
|  | 50 | 0.048 | 0.061 | 0.071 | 0.050 | 0.050 | 0.063 | 0.069 | 0.048 | 0.052 | 0.064 | 0.070 | 0.050 |
| **34** | 5 | 0.058 | 0.067 | 0.070 | 0.051 | 0.059 | 0.066 | 0.068 | 0.053 | 0.055 | 0.064 | 0.068 | 0.053 |
|  | 10 | 0.053 | 0.064 | 0.068 | 0.048 | 0.050 | 0.060 | 0.063 | 0.051 | 0.053 | 0.063 | 0.064 | 0.053 |
|  | 15 | 0.048 | 0.056 | 0.060 | 0.045 | 0.044 | 0.051 | 0.054 | 0.049 | 0.041 | 0.051 | 0.050 | 0.046 |
|  | 20 | 0.046 | 0.053 | 0.059 | 0.043 | 0.045 | 0.053 | 0.060 | 0.048 | 0.043 | 0.054 | 0.056 | 0.046 |
|  | 25 | 0.049 | 0.060 | 0.061 | 0.051 | 0.050 | 0.059 | 0.061 | 0.053 | 0.048 | 0.058 | 0.060 | 0.051 |
|  | 30 | 0.047 | 0.056 | 0.059 | 0.047 | 0.048 | 0.060 | 0.061 | 0.048 | 0.049 | 0.058 | 0.061 | 0.050 |
|  | 35 | 0.048 | 0.056 | 0.060 | 0.052 | 0.049 | 0.056 | 0.059 | 0.050 | 0.047 | 0.056 | 0.062 | 0.054 |
|  | 40 | 0.049 | 0.056 | 0.061 | 0.046 | 0.052 | 0.061 | 0.062 | 0.049 | 0.052 | 0.061 | 0.067 | 0.051 |
|  | 45 | 0.051 | 0.059 | 0.060 | 0.050 | 0.050 | 0.057 | 0.059 | 0.050 | 0.047 | 0.056 | 0.061 | 0.052 |
|  | 50 | 0.048 | 0.058 | 0.060 | 0.046 | 0.050 | 0.055 | 0.059 | 0.046 | 0.048 | 0.057 | 0.059 | 0.048 |
| **68** | 5 | 0.058 | 0.061 | 0.064 | 0.053 | 0.057 | 0.063 | 0.064 | 0.056 | 0.056 | 0.060 | 0.062 | 0.057 |
|  | 10 | 0.049 | 0.053 | 0.058 | 0.052 | 0.049 | 0.056 | 0.057 | 0.052 | 0.050 | 0.055 | 0.058 | 0.055 |
|  | 15 | 0.041 | 0.046 | 0.049 | 0.040 | 0.039 | 0.045 | 0.047 | 0.040 | 0.040 | 0.043 | 0.046 | 0.039 |
|  | 20 | 0.044 | 0.049 | 0.052 | 0.044 | 0.046 | 0.050 | 0.054 | 0.050 | 0.051 | 0.054 | 0.057 | 0.054 |
|  | 25 | 0.050 | 0.062 | 0.060 | 0.053 | 0.057 | 0.062 | 0.062 | 0.058 | 0.058 | 0.060 | 0.062 | 0.058 |
|  | 30 | 0.060 | 0.065 | 0.063 | 0.059 | 0.058 | 0.062 | 0.062 | 0.057 | 0.055 | 0.062 | 0.061 | 0.055 |
|  | 35 | 0.052 | 0.057 | 0.059 | 0.052 | 0.049 | 0.055 | 0.056 | 0.052 | 0.052 | 0.056 | 0.058 | 0.053 |
|  | 40 | 0.054 | 0.058 | 0.057 | 0.057 | 0.056 | 0.061 | 0.060 | 0.056 | 0.056 | 0.063 | 0.063 | 0.053 |
|  | 45 | 0.050 | 0.054 | 0.057 | 0.050 | 0.050 | 0.057 | 0.058 | 0.053 | 0.052 | 0.055 | 0.056 | 0.054 |
|  | 50 | 0.051 | 0.055 | 0.056 | 0.048 | 0.054 | 0.058 | 0.058 | 0.052 | 0.054 | 0.058 | 0.059 | 0.053 |

**Table A2: Results of the empirical power for the treatment effect=0.50 and stratum effect=0.50**

| **Number of clusters** | **Number of individuals per cluster** | **ICC=0.03** | | | | **ICC=0.06** | | | | **ICC=0.10** | | | |
| --- | --- | --- | --- | --- | --- | --- | --- | --- | --- | --- | --- | --- | --- |
|  |  | **CL Linear Regression** | **GEE** | **Meta-Regression** | **Mixed-effects** | **CL Linear Regression** | **GEE** | **Meta-Regression** | **Mixed-effects** | **CL Linear Regression** | **GEE** | **Meta-Regression** | **Mixed-effects** |
| **24** | 5 | 0.95 | 0.96 | 0.95 | 0.93 | 0.92 | 0.94 | 0.94 | 0.91 | 0.89 | 0.92 | 0.91 | 0.88 |
|  | 10 | 1.00 | 1.00 | 1.00 | 1.00 | 0.99 | 0.99 | 0.99 | 0.99 | 0.97 | 0.98 | 0.98 | 0.97 |
|  | 15 | 1.00 | 1.00 | 1.00 | 1.00 | 1.00 | 1.00 | 1.00 | 1.00 | 0.99 | 0.99 | 0.99 | 0.99 |
|  | 20 | 1.00 | 1.00 | 1.00 | 1.00 | 1.00 | 1.00 | 1.00 | 1.00 | 1.00 | 1.00 | 1.00 | 0.99 |
|  | 25 | 1.00 | 1.00 | 1.00 | 1.00 | 1.00 | 1.00 | 1.00 | 1.00 | 1.00 | 1.00 | 1.00 | 1.00 |
|  | 30 | 1.00 | 1.00 | 1.00 | 1.00 | 1.00 | 1.00 | 1.00 | 1.00 | 1.00 | 1.00 | 1.00 | 0.99 |
|  | 35 | 1.00 | 1.00 | 1.00 | 1.00 | 1.00 | 1.00 | 1.00 | 1.00 | 1.00 | 1.00 | 1.00 | 1.00 |
|  | 40 | 1.00 | 1.00 | 1.00 | 1.00 | 1.00 | 1.00 | 1.00 | 1.00 | 1.00 | 1.00 | 1.00 | 1.00 |
|  | 45 | 1.00 | 1.00 | 1.00 | 1.00 | 1.00 | 1.00 | 1.00 | 1.00 | 1.00 | 1.00 | 1.00 | 1.00 |
|  | 50 | 1.00 | 1.00 | 1.00 | 1.00 | 1.00 | 1.00 | 1.00 | 1.00 | 1.00 | 1.00 | 1.00 | 1.00 |
| **34** | 5 | 0.99 | 0.99 | 0.99 | 0.99 | 0.98 | 0.98 | 0.98 | 0.98 | 0.97 | 0.97 | 0.97 | 0.96 |
|  | 10 | 1.00 | 1.00 | 1.00 | 1.00 | 1.00 | 1.00 | 1.00 | 1.00 | 1.00 | 1.00 | 1.00 | 0.99 |
|  | 15 | 1.00 | 1.00 | 1.00 | 1.00 | 1.00 | 1.00 | 1.00 | 1.00 | 1.00 | 1.00 | 1.00 | 1.00 |
|  | 20 | 1.00 | 1.00 | 1.00 | 1.00 | 1.00 | 1.00 | 1.00 | 1.00 | 1.00 | 1.00 | 1.00 | 1.00 |
|  | 25 | 1.00 | 1.00 | 1.00 | 1.00 | 1.00 | 1.00 | 1.00 | 1.00 | 1.00 | 1.00 | 1.00 | 1.00 |
|  | 30 | 1.00 | 1.00 | 1.00 | 1.00 | 1.00 | 1.00 | 1.00 | 1.00 | 1.00 | 1.00 | 1.00 | 1.00 |
|  | 35 | 1.00 | 1.00 | 1.00 | 1.00 | 1.00 | 1.00 | 1.00 | 1.00 | 1.00 | 1.00 | 1.00 | 1.00 |
|  | 40 | 1.00 | 1.00 | 1.00 | 1.00 | 1.00 | 1.00 | 1.00 | 1.00 | 1.00 | 1.00 | 1.00 | 1.00 |
|  | 45 | 1.00 | 1.00 | 1.00 | 1.00 | 1.00 | 1.00 | 1.00 | 1.00 | 1.00 | 1.00 | 1.00 | 1.00 |
|  | 50 | 1.00 | 1.00 | 1.00 | 1.00 | 1.00 | 1.00 | 1.00 | 1.00 | 1.00 | 1.00 | 1.00 | 1.00 |
| **68** | 5 | 1.00 | 1.00 | 1.00 | 1.00 | 1.00 | 1.00 | 1.00 | 1.00 | 1.00 | 1.00 | 1.00 | 1.00 |
|  | 10 | 1.00 | 1.00 | 1.00 | 1.00 | 1.00 | 1.00 | 1.00 | 1.00 | 1.00 | 1.00 | 1.00 | 1.00 |
|  | 15 | 1.00 | 1.00 | 1.00 | 1.00 | 1.00 | 1.00 | 1.00 | 1.00 | 1.00 | 1.00 | 1.00 | 1.00 |
|  | 20 | 1.00 | 1.00 | 1.00 | 1.00 | 1.00 | 1.00 | 1.00 | 1.00 | 1.00 | 1.00 | 1.00 | 1.00 |
|  | 25 | 1.00 | 1.00 | 1.00 | 1.00 | 1.00 | 1.00 | 1.00 | 1.00 | 1.00 | 1.00 | 1.00 | 1.00 |
|  | 30 | 1.00 | 1.00 | 1.00 | 1.00 | 1.00 | 1.00 | 1.00 | 1.00 | 1.00 | 1.00 | 1.00 | 1.00 |
|  | 35 | 1.00 | 1.00 | 1.00 | 1.00 | 1.00 | 1.00 | 1.00 | 1.00 | 1.00 | 1.00 | 1.00 | 1.00 |
|  | 40 | 1.00 | 1.00 | 1.00 | 1.00 | 1.00 | 1.00 | 1.00 | 1.00 | 1.00 | 1.00 | 1.00 | 1.00 |
|  | 45 | 1.00 | 1.00 | 1.00 | 1.00 | 1.00 | 1.00 | 1.00 | 1.00 | 1.00 | 1.00 | 1.00 | 1.00 |
|  | 50 | 1.00 | 1.00 | 1.00 | 1.00 | 1.00 | 1.00 | 1.00 | 1.00 | 1.00 | 1.00 | 1.00 | 1.00 |

**Table A3: Results of the average standard error for the treatment effect=0.11 and stratum effect=0.11**

| **Number of clusters** | **Number of individuals per cluster** | **ICC=0.03** | | | | **ICC=0.06** | | | | **ICC=0.10** | | | |
| --- | --- | --- | --- | --- | --- | --- | --- | --- | --- | --- | --- | --- | --- |
|  |  | **CL Linear Regression** | **GEE** | **Meta-Regression** | **Mixed-effects** | **CL Linear Regression** | **GEE** | **Meta-Regression** | **Mixed-effects** | **CL Linear Regression** | **GEE** | **Meta-Regression** | **Mixed-effects** |
| **24** | 5 | 0.14 | 0.13 | 0.16 | 0.14 | 0.14 | 0.14 | 0.14 | 0.15 | 0.15 | 0.15 | 0.15 | 0.15 |
|  | 10 | 0.10 | 0.10 | 0.12 | 0.10 | 0.11 | 0.11 | 0.11 | 0.11 | 0.13 | 0.12 | 0.12 | 0.12 |
|  | 15 | 0.09 | 0.09 | 0.10 | 0.09 | 0.10 | 0.10 | 0.10 | 0.10 | 0.11 | 0.11 | 0.11 | 0.11 |
|  | 20 | 0.08 | 0.08 | 0.09 | 0.08 | 0.09 | 0.09 | 0.09 | 0.09 | 0.11 | 0.11 | 0.11 | 0.11 |
|  | 25 | 0.08 | 0.07 | 0.09 | 0.08 | 0.09 | 0.09 | 0.09 | 0.09 | 0.11 | 0.10 | 0.10 | 0.10 |
|  | 30 | 0.07 | 0.07 | 0.08 | 0.07 | 0.09 | 0.08 | 0.08 | 0.09 | 0.10 | 0.10 | 0.10 | 0.10 |
|  | 40 | 0.07 | 0.06 | 0.08 | 0.07 | 0.08 | 0.08 | 0.08 | 0.08 | 0.10 | 0.10 | 0.10 | 0.10 |
|  | 45 | 0.07 | 0.06 | 0.08 | 0.06 | 0.08 | 0.08 | 0.08 | 0.08 | 0.10 | 0.10 | 0.10 | 0.10 |
|  | 50 | 0.06 | 0.06 | 0.07 | 0.06 | 0.08 | 0.08 | 0.08 | 0.08 | 0.10 | 0.09 | 0.10 | 0.10 |
| **34** | 5 | 0.11 | 0.11 | 0.11 | 0.12 | 0.12 | 0.12 | 0.12 | 0.12 | 0.13 | 0.13 | 0.13 | 0.13 |
|  | 10 | 0.09 | 0.08 | 0.08 | 0.09 | 0.09 | 0.09 | 0.09 | 0.09 | 0.11 | 0.10 | 0.10 | 0.10 |
|  | 15 | 0.07 | 0.07 | 0.07 | 0.07 | 0.08 | 0.08 | 0.08 | 0.08 | 0.10 | 0.09 | 0.10 | 0.10 |
|  | 20 | 0.07 | 0.07 | 0.07 | 0.07 | 0.08 | 0.08 | 0.08 | 0.08 | 0.09 | 0.09 | 0.09 | 0.09 |
|  | 25 | 0.06 | 0.06 | 0.06 | 0.06 | 0.08 | 0.07 | 0.07 | 0.08 | 0.09 | 0.09 | 0.09 | 0.09 |
|  | 30 | 0.06 | 0.06 | 0.06 | 0.06 | 0.07 | 0.07 | 0.07 | 0.07 | 0.09 | 0.09 | 0.09 | 0.09 |
|  | 35 | 0.06 | 0.06 | 0.06 | 0.06 | 0.07 | 0.07 | 0.07 | 0.07 | 0.09 | 0.08 | 0.08 | 0.09 |
|  | 40 | 0.06 | 0.05 | 0.06 | 0.06 | 0.07 | 0.07 | 0.07 | 0.07 | 0.08 | 0.08 | 0.08 | 0.08 |
|  | 45 | 0.06 | 0.05 | 0.05 | 0.05 | 0.07 | 0.07 | 0.07 | 0.07 | 0.08 | 0.08 | 0.08 | 0.08 |
|  | 50 | 0.05 | 0.05 | 0.05 | 0.05 | 0.07 | 0.07 | 0.07 | 0.07 | 0.08 | 0.08 | 0.08 | 0.08 |
| **68** | 5 | 0.08 | 0.08 | 0.08 | 0.08 | 0.09 | 0.08 | 0.08 | 0.09 | 0.09 | 0.09 | 0.09 | 0.09 |
|  | 10 | 0.06 | 0.06 | 0.06 | 0.06 | 0.07 | 0.07 | 0.07 | 0.07 | 0.07 | 0.07 | 0.07 | 0.07 |
|  | 15 | 0.05 | 0.05 | 0.05 | 0.05 | 0.06 | 0.06 | 0.06 | 0.06 | 0.07 | 0.07 | 0.07 | 0.07 |
|  | 20 | 0.05 | 0.05 | 0.05 | 0.05 | 0.06 | 0.06 | 0.06 | 0.06 | 0.07 | 0.06 | 0.06 | 0.07 |
|  | 25 | 0.04 | 0.04 | 0.04 | 0.04 | 0.05 | 0.05 | 0.05 | 0.05 | 0.06 | 0.06 | 0.06 | 0.06 |
|  | 30 | 0.04 | 0.04 | 0.04 | 0.04 | 0.05 | 0.05 | 0.05 | 0.05 | 0.06 | 0.06 | 0.06 | 0.06 |
|  | 35 | 0.04 | 0.04 | 0.04 | 0.04 | 0.05 | 0.05 | 0.05 | 0.05 | 0.06 | 0.06 | 0.06 | 0.06 |
|  | 40 | 0.04 | 0.04 | 0.04 | 0.04 | 0.05 | 0.05 | 0.05 | 0.05 | 0.06 | 0.06 | 0.06 | 0.06 |
|  | 45 | 0.04 | 0.04 | 0.04 | 0.04 | 0.05 | 0.05 | 0.05 | 0.05 | 0.06 | 0.06 | 0.06 | 0.06 |
|  | 50 | 0.04 | 0.04 | 0.04 | 0.04 | 0.05 | 0.05 | 0.05 | 0.05 | 0.06 | 0.06 | 0.06 | 0.06 |
